# From importation to autochthonous transmission: Drivers of chikungunya and dengue emergence in a temperate area

**DOI:** 10.1371/journal.pntd.0008320

**Published:** 2020-05-11

**Authors:** Frédéric Jourdain, David Roiz, Henriette de Valk, Harold Noël, Grégory L’Ambert, Florian Franke, Marie-Claire Paty, Anne Guinard, Jean-Claude Desenclos, Benjamin Roche

**Affiliations:** 1 Santé publique France (French National Public Health Agency), Saint-Maurice, France; 2 MIVEGEC Unit, IRD 224, CNRS 5290, Univ Montpellier, Montpellier, France; 3 Entente interdépartementale pour la démoustication du littoral méditerranéen (EID Méditerranée), Montpellier, France; 4 Santé publique France (French National Public Health Agency), Marseille, France; 5 Santé publique France (French National Public Health Agency), Toulouse, France; Institut Pasteur, FRANCE

## Abstract

**Background:**

The global spread of *Aedes albopictus* has exposed new geographical areas to the risk of dengue and chikungunya virus transmission. Several autochthonous transmission events have occurred in recent decades in Southern Europe and many indicators suggest that it will become more frequent in this region in the future. Environmental, socioeconomic and climatic factors are generally considered to trigger the emergence of these viruses. Accordingly, a greater knowledge of the determinants of this emergence in a European context is necessary to develop adapted surveillance and control strategies, and public health interventions.

**Methodology/Principal findings:**

Using French surveillance data collected from between 2010 and 2018 in areas of Southern France where *Ae*. *albopictus* is already established, we assessed factors associated with the autochthonous transmission of dengue and chikungunya. Cases leading to autochthonous transmission were compared with those without subsequent transmission using binomial regression. We identified a long reporting delay (≥ 21 days) of imported cases to local health authorities as the main driver for autochthonous transmission of dengue and chikungunya in Southern France. The presence of wooded areas around the cases’ place of residence and the accumulation of heat during the season also increased the risk of autochthonous arbovirus transmission.

**Conclusions:**

Our findings could inform policy-makers when developing strategies to the emerging threats of dengue and chikungunya in Southern Europe and can be extrapolated in this area to other viruses such as Zika and yellow fever, which share the same vector. Furthermore, our results allow a more accurate characterization of the environments most at risk, and highlight the importance of implementing surveillance systems which ensure the timely reporting and of imported cases and swift interventions.

## Author summary

The dengue, chikungunya and Zika viruses have tremendously expanded their geographic range during recent decades and are now considered emerging threats in temperate areas. The increase in international travel and trade appear to be major factors, encouraging both a circulation of these viruses on a global scale and the dispersion of one of their main vectors: *Aedes albopictus*, the tiger mosquito. However, these two factors cannot fully explain the risk of viral emergence in temperate areas. Climatic factors, land cover, socioeconomic characteristics and public health systems also likely impact the risk of virus transmission. In this context, we performed a statistical analysis of the different imported cases of dengue and chikungunya in Southern France, where the tiger mosquito is already established. A comparison between contexts with local virus transmission and situations without local transmission made it possible to identify the factors associated with the risk of viral circulation. We found that a delay in reporting imported cases, the presence of wooded areas around the cases’ places of residence, and the accumulation of heat during the season, all increased the risk of transmission. The identification of these conditions is therefore of primary importance both to determine the risk of the emergence of these viruses, and to improve public health policies.

## Introduction

The dengue (DENV) and chikungunya (CHIKV) viruses have greatly expanded their geographic range globally in recent decades [1] and are considered emerging public health threats throughout the world, including Europe [2]. The global number of dengue infections in 2010 was estimated at 390 (284–528) million per year, 96 (67–136) million cases being clinically manifested [3]. CHIKV has been responsible for two major epidemics in recent decades. The first spread in 2004 from Eastern Africa to the Indian Ocean and to South Asia. The second occurred in the Americas, with more than 1.2 million suspected cases reported for the 2013–2014 period [4]. DENV and CHIKV are mainly transmitted between humans through the bite of *Aedes aegypti* and *Aedes albopictus* mosquitoes in urban settings, and are introduced in non-endemic countries by infected returning travellers [5,6]. Autochthonous transmission can then occur in areas where a competent vector is established and where climatic conditions are favourable for transmission. In the Mediterranean and central Europe, only *Ae*. *albopictus* is present. Its expansion is a direct consequence of the globalization of trade [7]. The continued spread of this vector through trade and the constant growth in international travel will increase the risk of exotic viruses emerging in many other European areas. Italy, France, Croatia and Spain experienced several events of autochthonous DENV and CHIKV transmission between 2010 and 2018 [8–18]. Nevertheless, the number of imported cases remains well above the number of autochthonous transmission cases [19] and, to date, there is no evidence-based explanation as to why autochthonous transmission occurs in some circumstances in Europe but not in others.

While the presence of an established vector population and virus introduction by infected travellers are necessary conditions for the emergence of these infections, they may not be sufficient for arbovirus transmission. Indeed, effective transmission is multifactorial and results from complex interactions between mosquito vectors, the human population, viral agents, their environment and climate. Genetics play an important role in fostering the transmission of some viral genotypes by locally established vector populations [21,22]. Socioeconomic and environmental factors influence the epidemiology of the disease by affecting the introduction of the virus, the contact between vectors and hosts, vector-pathogen interactions, as well as vector population distribution and dynamics [3,23–25]. Finally, public health interventions are likely to alter the dynamics of infection transmission [26].

*Aedes albopictus* became established in France in 2004 and has since spread throughout a large part of the country [20]. The French population can be considered fully susceptible to DENV and CHIKV infection. There is no specific antiviral drug treatment or recommended vaccine in France for DENV or CHIKV infection. Therefore, prevention and control of these infections is based on i) larval control to reduce the vector population as a preventive measure, ii) the surveillance of human imported cases, iii) the early detection of any local transmission, and iv) the implementation of proportionate vector control measures to prevent and contain autochthonous transmission [27]. A national preparedness and response plan has been implemented since 2006 [28].

Few autochthonous transmission events have occurred in France in recent years [8,13–18] but the situation continues to evolve. *Aedes albopictus* is still spreading across the country, leading to a greater proportion of the population exposed to DENV and CHIKV transmission risk. Other emerging viruses may also prove to be a challenge for the country’s preparedness and response systems, something already observed with the Zika virus when cases were reported in France in 2016 following the epidemic in the Americas [29]. Although substantial human and logistical resources are already mobilized every year, the challenges raised by emerging viruses will only lead to further costs and this raises the question of the future sustainability of France’s surveillance and control system [19]. This imminent situation underlines the need for a better understanding of the factors that favour DENV and CHIKV autochthonous transmission in France.

Several statistical and mathematical models have been used to identify the determinants of the distribution and abundance of *Ae*. *albopictus*, as well as the associated transmission risk of DENV and CHIKV [30–34]. Whereas contexts where transmission is high have been widely investigated (especially for DENV), the number of studies in places with sporadic and limited transmission remain scarce [35–37].

The present study aimed to identify and quantify the relative importance of the factors associated with DENV and CHIKV autochthonous transmission events in mainland France following the introduction of a viremic traveller in areas where *Ae*. *albopictus* is already established. We based our analysis on the enhanced surveillance of imported cases and performed an in-depth study of the different transmission events identified in France during recent years by the national arbovirosis surveillance system. This work was carried out within the context of improving the country’s preparedness and response system for arboviral risks in temperate areas.

## Materials and methods

Binomial regression was used to compare cases leading to autochthonous transmission with those which had no subsequent transmission.

### Case definitions

The different case definitions adopted in the study are indicated in Table 1. An autochthonous transmission event was defined as the occurrence of at least one autochthonous case in the study area and during the study period. The duration of viremia was fixed at 10 days: corresponding to a period between two days before the date of symptom onset and 7 days after this date [43,44]. Only cases present in an area colonized by *Ae*. *albopictus* during their viremia were included.

**Table 1:** Case definitions for dengue and chikungunya virus infection.

Definition	Dengue	Chikungunya
**Suspected case**	Fever of 38.5°C or higher and at least one of the following symptoms not explained by other medical conditions: headache, back pain, retro-orbital pain, myalgia, and arthralgia.	
**Probable case**	Suspected case with positive IgM antibodies in a single sample	
**Confirmed case**	Suspected case with at least one of the following biological results: - Positive RT-PCR - seroconversion - positive NS1 test - 4-fold increase in IgG antibodies	Suspected case with at least one of the following biological results: - Positive RT-PCR - seroconversion - 4-fold increase in IgG antibodies
**Imported case**	Case with travel history in the 15 days before the onset of symptoms in an area known for DENV or CHIKV circulation	
**Autochthonous case**	Case without travel history in the 15 days before the onset of symptoms in an area known for DENV or CHIKV circulation	

### Study area and period

The study area comprises the following five French administrative districts (*départements* in French) (with European NUTS 3 statistical classification [38]) along the Mediterranean coast colonized by *Ae*. *albopictus*: Alpes-Maritimes, Var, Bouches-du-Rhône, Gard and Hérault. It covers a total area of 27 436 km^2^ with a population of 6.1 million people at the end of 2018 [39].

**Fig 1:**
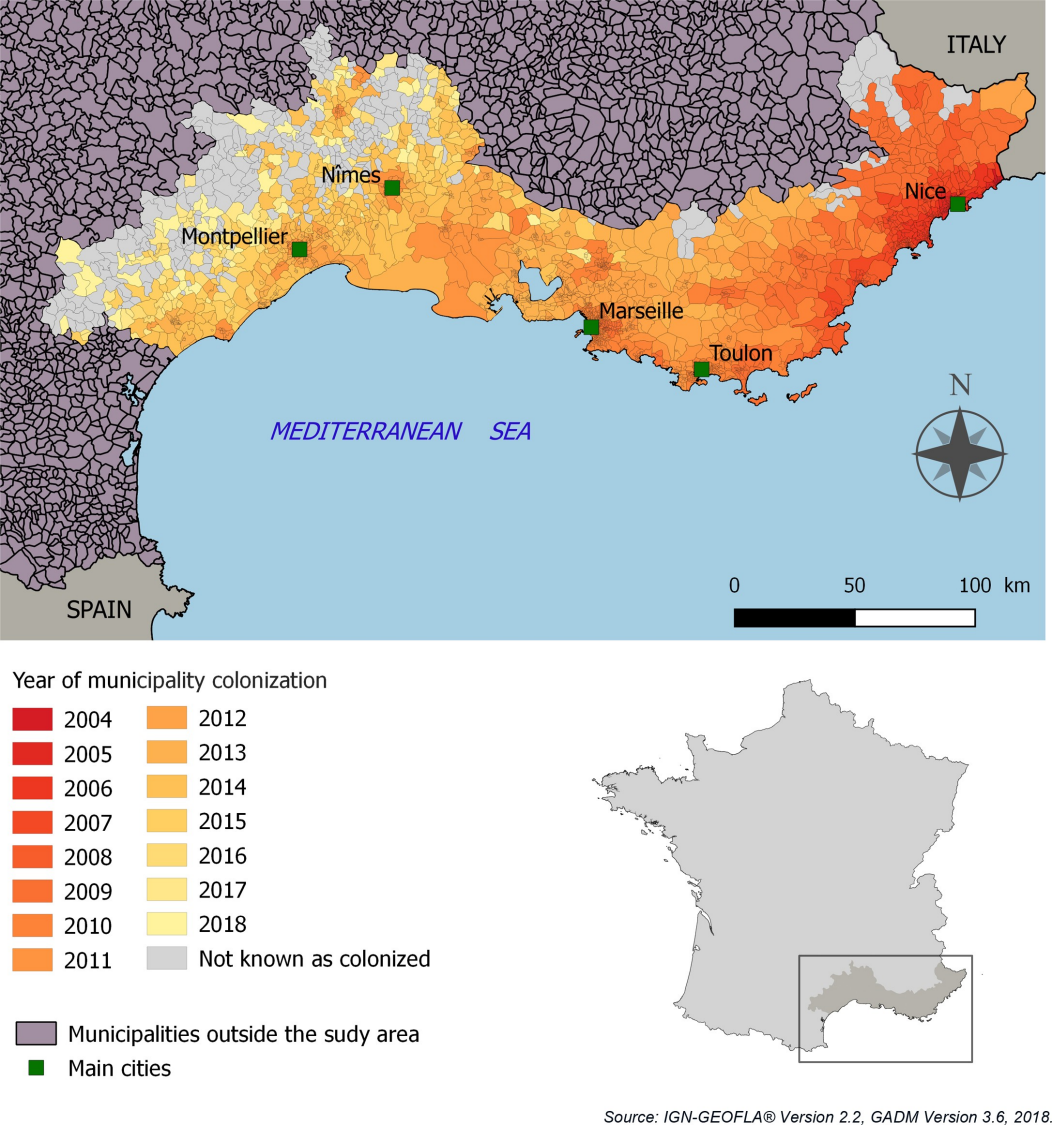
Spatial and temporal distribution of *Aedes albopictus* in the study area. Source of data: national surveillance of invasive mosquitoes, EID-Méditerranée.

This area has the oldest recorded *Ae*. *albopictus* establishment in France, with progressive colonization from East to West since 2004 (Fig 1). In 2019, the invasive process is still ongoing in the western and northern parts of the study area. The area is characterized by a typically Mediterranean climate in the coastal region, with dry summers, mild winters and irregular rainfall concentrated mainly in autumn, and potential spring downpours [40].

The study period included the season from 1 May through 30 November—representing the seasonal activity of *Ae*. *albopictus* in the South of France [41]–for the years 2010, 2013, 2014, 2015, 2017 and 2018. The three years not included were due to the lack of epidemiological (2012) and entomological data (2011, 2016). Our study period included all the years when an autochthonous event was observed (Table 2).

**Table 2:** Dengue and chikungunya autochthonous transmission events in France between 2010 and 2018.

Year	Locality	Département	Virus	Number of autochthonous cases	Identification of the imported source case	Ref.
**2010**	Nice	Alpes-Maritimes	Dengue 1	2	Yes	[18]
**2010**	Fréjus	Var	Chikungunya	2	Yes	[17]
**2011**		*Absence of autochthonous transmission event*				
**2012**		*Absence of autochthonous transmission event*				
**2013**	Venelles	Bouches-du-Rhône	Dengue 2	1	Yes	[16]
**2014**	Aubagne	Bouches-du-Rhône	Dengue 2	2	Yes	[42]
**2014**	Toulon	Var	Dengue 1	1	No	[42]
**2014**	Toulon	Var	Dengue 2	1	No	[42]
**2014**	Montpellier	Hérault	Chikungunya	12	Yes	[15]
**2015**	Nîmes	Gard	Dengue 1	7	Yes	[14]
**2016**		*Absence of autochthonous transmission event*				
**2017**	Le Cannet-des-maures (*)	Var	Chikungunya	11	Yes	[13]
**2017**	Taradeau (*)	Var	Chikungunya	6	Yes	[13]
**2018**	Saint-Laurent-du-Var	Alpes-Maritimes	Dengue 2	5	No	[8]
**2018**	Nîmes	Gard	Dengue 1	1	Yes	[8]
**2018**	Clapiers	Hérault	Dengue 1	2	No	[8]

(*) These transmission foci were geographically separate but an epidemiological link was established.

### Data sources

Data sources which had both with nationwide coverage and regular updates were preferred. The different data sources are summarized in Table 3 and detailed in the following sections.

**Table 3:** Data sources.

Data type	Source
Epidemiological	Data from the national arbovirus surveillance, Santé publique France (French Public Health Agency) https://www.santepubliquefrance.fr/
Vector control interventions	Entente Interdépartementale pour la démoustication du littoral méditerranéen—Interdepartmental Agency for mosquito control on the Mediterranean coast, (EID Méditerranée) http://www.eid-med.org/
Rainfall and temperature	French meteorological agency. Météo France http://www.meteofrance.com/accueil
NDVI	Landsat 7 and Landsat 8 data processed by the THEIA Land Data Centre (surface reflectance corrected for atmospheric effects and cloud cover level: Level 2A) https://theia.cnes.fr/ based on images acquired by the United States Geological Survey (USGS)
Land cover	Land cover map produced by the Center for the Study of the Biosphere from Space (CESBIO), THEIA Land Data Centre http://www.cesbio.ups-tlse.fr/index_us.htm
Housing and vegetation	BD TOPO, Institut national de l’information géographique et forestière http://www.ign.fr/
Demographic	French National Institute of Statistics and Economic Studies (INSEE) https://www.insee.fr/en/accueil
Socioeconomic	French National Institute of Statistics and Economic Studies (INSEE) https://www.insee.fr/en/accueil
Social Deprivation index	French National Institute for Health and Medical Research (Inserm) https://geo.data.gouv.fr/fr/datasets/9c6009a2bb10c4d69a15d399def4770b038be18a

#### Epidemiological data

Dengue and chikungunya case data were obtained through the national surveillance system for arbovirosis, which comprises active human surveillance based on the reporting of suspected cases of dengue and/or chikungunya to public health authorities, followed by timely biological diagnosis. The human surveillance system was previously described by Paty and coll. [19]. Only confirmed or probable imported cases were included in the present analysis. We used the earliest date of presence of the case during viremia (EDP) in the study area to calculate the different delays (i.e., sampling, reporting, etc.). EDP could be either the date of symptom onset or the return date from travel (in the case of symptom onset outside the study area). The sampling delay was defined as the period of time between EDP and the first date of blood sampling for biological testing. The reporting delay was defined as the period of time between EDP and the moment health authorities received report of the case. The exposure duration was defined as the time interval between EDP and the end of viremia of the imported case. Imported cases arriving in the study area 7 days or more after the date of symptom onset were excluded since they were considered to be no longer viremic.

#### Vector control interventions data

Each observation was completed with data derived from details regarding entomological operations (entomological survey, vector control). Data were obtained from the French mosquito control agency, EID-Méditerranée. The intervention delay was defined as the period of time between EDP and the first intervention focusing on vectors, whether an entomological survey or vector control. Entomological surveys aim to identify the presence of the vector–pre-imaginal stages or adults—while control measures are taken when the vector’s presence is confirmed. Vector control is implemented according to national guidelines and depends on the local entomological, epidemiological and environmental context. The intervention may consist of one or more of the following components: source reduction of breeding sites, application of larvicides, hand-held thermal fogging and vehicle-mounted ultra-low volume fogging. *Bacillus thuringiensis israelensis* and diflubenzuron are used as larvicides while deltamethrin is the main sprayed adulticide.

#### Social, environmental and meteorological data

Observations were georeferenced within the French national address database https://adresse.data.gouv.fr/) depending on the available information. When a specific address was not available, the localization of an observation was characterized at the municipality level within the specific *département*. Social, environmental and meteorological explanatory variables were selected according to results from other studies in the literature [30,45–47].

Socioeconomic and demographic data were locally obtained at the IRIS Census unit level for georeferenced observations. IRIS are aggregated units used in France for statistical purposes. They constitute the smallest geographical unit for which population census data with housing and socioeconomic details are available in France. They are defined with a target size of 2000 inhabitants per basic unit and data for each IRIS are supplied by the French National Institute of Statistics and Economic Studies (INSEE, www.insee.fr). They provide information on the following variables: the characteristics of each household and of the persons who compose that household (marital status, activity/inactivity, socio-professional category), the characteristics of each family (number of children), the use of residences (principal residences, secondary residences, vacant) as well as the proportions of the type of dwelling (houses *versus* apartments). We used the French social deprivation index (FDep09), a socioeconomic indicator which is available at the IRIS scale [48], to assess the influence of social inequity.

Normalized difference vegetation indexes (NDVI) were derived from Landsat 7 and Landsat 8 satellite imagery data (level 2A) processed by the THEIA Land Data Centre. For each georeferenced observation, an image was selected for NDVI calculation, with the image shooting date as close as possible to the date of symptom onset of the case and with cloud cover less than 10%. Mean NDVI were calculated within buffers of 300m around each geo-located observation.

Daily meteorological measurements (precipitation, minimal and maximal temperature, diurnal temperature range) were obtained from the French meteorological agency (Météo France) for the period from 1 March to 30 November of each study year. For each observation, the closest meteorological station was selected from among the 147 stations located within or in the direct vicinity of the study area. The average distance between case location and the associated meteorological station was 6.4 km (sd = 3.6 km) for temperature data and 6.2 km (sd = 3.6) for rainfall data. Mean (T_mean_), maximal (T_max_) and minimal (T_min_) temperatures were computed for the 7 and 10 days before and after EDP, respectively. A bounded accumulated Growing Degree Days (GDD) index was also calculated with a baseline temperature of 11°C and a maximum threshold of 1350°C as proposed by Roiz *et al*. [36] at EDP (GDD_0_) and 10 days after EDP (GDD_10_). Different time windows were considered for calculation of weekly accumulated rainfall in the 1 to 4 weeks before EDP. Weekly diurnal temperature ranges (DTR) were also computed after EDP. A period of 7 to 10 days was chosen for the construction of different temporal variables, as this period corresponds to the duration proposed in the literature for the extrinsic incubation of DENV and CHIKV in *Ae*. *albopictus* [49].

Land cover data were extracted from a map produced from optical imaging by the THEIA Land Data Centre [50]. This map is available at the national scale with 17 land cover classes and 10 m spatial resolution. Housing and vegetation were also characterized with the BD TOPO produced by the French National Geographic Institute [51]. Land cover information was extracted in buffers of different sizes, i.e. 100 m, 200 m and 300 m radius zones. This range of buffers was chosen according to existing knowledge on *Ae*. *albopictus* dispersal [52].

### Statistical analysis

To identify factors associated with the emergence of autochthonous transmission events of DENV and CHIKV in mainland France, we based our analysis on all imported cases identified by the national surveillance system within the study area during the study period. For every imported case, we considered each place where the case was present for at least an hour during viremia as an observation. This implies that a single notified case could result in more than one observation, depending upon the case’s movements and the number of places where the case stayed during the infectious period. Only observations in localities known to be colonized by *Ae*. *albopictus* during stays of notified cases were included.

Finally, we compared observations that led to an autochthonous transmission event (n = 13) with observations which did not (n = 844) in order to assess the influence of the above possible explanatory variables on the occurrence of dengue and chikungunya autochthonous events. Comparison was made using complementary log-log regression. The binary response variable of interest was therefore the occurrence of an autochthonous event, coded as “1” in the case of autochthonous transmission and coded as “0” in the absence of autochthonous transmission. We used a generalized linear model with a ‘complementary log-log’ link function, as the probability of the event occurring appeared small.

**Fig 2:**
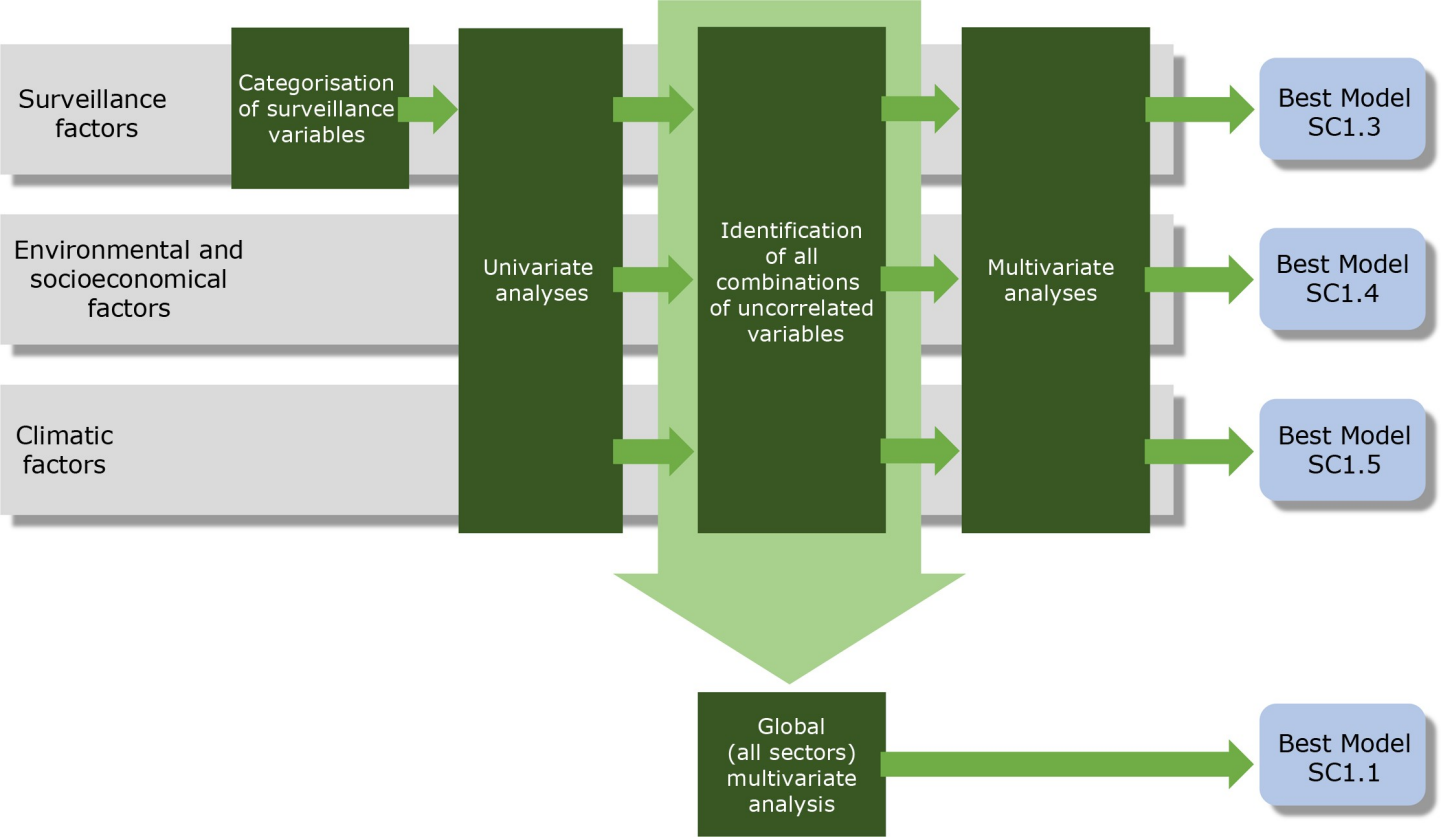
General outline of the analysis strategy.

Univariate binomial regression models were first used to identify candidate variables for the multivariate analysis, while avoiding collinearity. Variables with a *p*-value <0.25 (arbitrarily chosen) in the univariate analyses were selected as candidates for the multivariate analysis. Spearman’s correlation coefficient was calculated for pairwise variables. Based on the correlation analysis, different sets of variables were defined to ensure that collinearity was reduced. Multivariate models were then built based on univariate analyses using the various sets of variables that did not have statistically significant pairwise correlations. Variance Inflation Factors (VIF) were used to assess multicollinearity between selected variables [53]. Prior to performing a global multivariate analysis, different multivariate models were built for each type of factor (surveillance, climatic, socioeconomic and environmental factors). These analyses (hereinafter referred to as “multivariate sectoral analyses”) were performed to assess the variance explained by each category of variable and to take into account the difference in the number of observations for each type of factor. Variables were selected for multivariate analyses using forward and backward selection. Best-fit models were selected on the basis of Akaike Information Criteria. Statistical analyses was performed using R software [54] with MASS [55] and MuMIn packages [56] for model selection. A general diagram of the analysis strategy is shown in Fig 2.

### Management of missing data

Social, surveillance, environmental and meteorological data were missing for some observations as no imported cases were identified for four events of autochthonous dengue transmission (Table 2). Two of these four events led to more than one autochthonous case. For these clusters, a likely place of transmission was identified and the associated focal point of transmission consequently set at the centre of the transmission area. This hypothesis seems reasonable since all clusters occurred in an area with a radius of less than 300 meters. For events with only one autochthonous case, two scenarios were formulated, the first (Sc1) at the residence of the autochthonous case, while the second (Sc2) considered the absence of georeferenced information. For meteorological data, the time of introduction was set at 30 days prior to the date of symptoms onset of the first autochthonous case. This period was chosen as it is the mean time interval between the occurrence of the first autochthonous case for the five dengue transmission events for which an imported index case was identified and the date of return of imported primary dengue cases (n = 5, Table 2). We only relied on dengue cases as this situation (i.e., a single autochthonous case in the absence of any identified imported case) only occurred for dengue.

We then considered different options (detailed below) to assess the model's response to variations in the “reporting delay” variable. A threshold in the reporting delay was defined arbitrarily at 21 days. This duration corresponds to the average cycle of virus transmission from the mosquito infective blood meal to the end of viremia in the first autochthonous human case. We considered the following options:

- Option 1: only values recorded prior to the identification of the autochthonous focus retained;
- Option 2: values recorded after identification of the outbreak introduced;
- Option 3: all missing values and delays exceeding 21 days set at 21 days;
- Option 4: this scenario is similar to that of scenario 3, but the variable is converted to a categorical variable. Delay during [0;21 [set as “short to medium” and delay ≥ 21 days as “long”;
- Option 5: all missing data (including reports of imported cases after autochthonous circulation) classified in a dedicated group, “missing” group.

These different options were considered using univariate analysis to select the one that would be retained in subsequent multivariate analyses.

### Ethics statements

This study was part of French national public health surveillance program for vector-borne diseases at Santé publique France (the French Agency for Public Health), a governmental agency reporting to the French Ministry of Health. All data were anonymized. Data collection through the epidemiological surveillance system was approved by the French Commission on Information Technology and Liberties (CNIL), with authorization n°911185.

## Results

A total of 857 observations were included in the different analyses. This number corresponds to imported cases who met all four of the following criteria: (i) stayed for more than an hour in areas where the vector was present during the time they were there, (ii) were still viremic, (iii) were classified either as a confirmed or probable case, and (iv) had at least one spatial indication of stay at the municipality level.

### Univariate analyses

Twenty-seven variables with a p value < 0.25 (Table 4) in the univariate analyses were selected for multivariate analysis (S1 Table).

**Table 4:** Results of the univariate binomial regression analyses sorted according to their p-values.

Variable	Type^(1)^	D^2^	p-value	OR (95% CI)
T_mean_ 7 days before EDP (T_mean_7)	M	8.8%	0.0013	1.395 (1.148–1.723)
T_min_ 7 days before EDP (T_min_7)	M	7.4%	0.0022	1.346 (1.115–1.642)
Reporting delay (RD)	SC	7.6%	0.0024	1.042 (1.008–1.065)
Intervention delay	SC	9.6%	0.0026	1.068 (1.014–1.111)
T_max_ 7 days before EDP (T_max_7)	M	6.9%	0.0032	1.264 (1.086–1.483)
T_max_ 10 days after EDP (T_max_10)	M	6.5%	0.0047	1.273 (1.082–1.507)
T_mean_ 10 days after EDP (T_mean_10)	M	6.2%	0.0073	1.345 (1.094–1.683)
Discontinuous urban fabrics within a radius of 100 m (DUF_100_)	LC	6.9%	0.0077	1.011 (1.004–1.020)
Percentage of “house”- type residences at the IRIS scale	SE	5.7%	0.0103	11.42 (1.96–88.26)
Percentage of vegetation within a radius of 300 m (Vegetation)	LC	3.4%	0.0175	1.033 (1.003–1.058)
Bounded growing degree days 10 days after EDP (GDD_10_)	M	6.4%	0.0181	1.003 (1.001–1.005)
Sampling delay	SC	4.7%	0.0185	1.037 (0.993–1.061)
Bounded growing degree days until EDP (GDD_0_)	M	5.8%	0.0203	1.002 (1.001–1.005)
Discontinuous urban fabrics within a radius of 300 m (DUF_300_)	LC	3.8%	0.0332	1.001 (1.000–1.002)
T_min_ 10 days after EDP (T_min_10)	M	3.3%	0.0415	1.214 (1.012–1.470)
Percentage of families in households	SE	3.2%	0.0582	68.25 (1.21–8364.41)
NDVI within a radius of 300 m	M/LC	2.5%	0.0074	93.15 (0.70–14501.45)
Diurnal temperature range (DTR)	M	2.1%	0.0894	1.185 (0.973–1.444)
Day of the year	M	2.1%	0.0969	1.010 (0.998–1.023)
Weekly rainfall 3 weeks before EDP (Rain_3w_)	M	1.1%	0.1195	1.009 (0.992–1.017)
Percentage of main residences (Main Res.)	SE	2.7%	0.1212	210.5 (0.8–597711.4)
Weekly rainfall 2 weeks before EDP (Rain_2w_)	M	2.9%	0.1515	0.938 (0.833–1.000)
Percentage of vacant residences	SE	1.8%	0.1595	0.000 (0.000–14.634)
Number of buildings within a radius of 300 m (Buildings)	LC	2.0%	0.1610	0.999 (0.997–1.000)
Length of viremia (days) in the study area	SC	2.4%	0.1869	1.301 (0.942–2.131)
Continuous urban fabric within a radius of 300 m	LC	2.6%	0.2000	0.998 (0.993–1.000)
Weekly rainfall 1 week before EDP (Rain_1w_)	M	1.9%	0.2287	0.949 (0.844–1.008)

Only variables below the selected threshold are presented. D^2^: explained deviance.

DUF: discontinuous urban fabrics (between 30 to 80% of the total surface is impermeable, covered by buildings, roads and artificially surfaced areas); EDP: earliest date of presence of an imported case during viremia in the study area; GDD: bounded growing degree days; NDVI: normalized difference vegetation index.

(1) The different variables are classified as follows. LC: variable related to land cover. S: variable related to surveillance and control. SE: socioeconomic variable. M: meteorological variable. RD: reporting delay (in days), as the period between the earliest date of presence of an imported case and the date of case reporting.

### Multivariate analyses

Collinearity was mainly present due to the construction of the variables to be studied (e.g., temperatures and GDD with different time steps or land cover characteristics with different buffers). The different variables linked to surveillance and response activities (i.e., “Reporting delay”, “Sampling delay” and “intervention delay”) were strongly correlated. Considering variables linked to surveillance and response, using reporting delay instead of sampling delay or intervention delay resulted in a lower Aikake Information Criterion (AIC) and more explained deviance. In subsequent analyses we therefore chose to focus on the reporting delay (RD). Moreover, the RD reflects the time required for a case to be registered in the public health system and constitutes an operational reality.

Option 5 was selected for the transformation of the RD variable. The RD was therefore converted into a categorical variable (RDC) for the subsequent analyses as (1) it exhibited the lowest AIC score among comparable models, (2) had high explained deviance, and (4) allowed us to keep the entire dataset without imputation of missing data for transmission events. All the results of the binomial regression univariate model for different options of the RD on the risk of autochthonous arbovirus transmission are shown in S1 Appendix.

Different models were therefore built by combination of uncorrelated variables. For all the different models, the VIF for each predictor was less than 3, which is the value proposed as a threshold below which any effect of multicollinearity can be considered negligible [57]. Global multivariate models were built as a final step by combining the different type of variables with the reporting delay converted as a categorical variable. Identical global multivariate models were obtained after stepwise selection for scenarios Sc1 and Sc2. However, global multivariate models for scenario Sc1 (scenario for which the transmission location is fixed at the residence of the autochthonous case) exhibits the highest explained deviance. The different models obtained for scenario Sc1 are presented in Table 5. Models obtained for scenario Sc2 are shown in S2 Table.

**Table 5:** Results of binomial regression of emergence of autochthonous arbovirus infections for scenario Sc1 (place of transmission at the residence of the autochthonous case).

Model	Variables	Variable category	df	logLik	AICc	delta	weight	D^2^
	Global multivariate models							
Sc1.1	RD, Vegetation, GDD_0_	All	5	-24.945	59.97	0.00	0.68	62.2%
Sc1.2	RD, Vegetation, GDD_10_	All	5	-25.745	61.57	1.60	0.31	61.0%
	Multivariate sectoral models							
Sc1.3	RD	S	3	-31.204	68.44	8.47	0.010	52.8%
Sc1.4	Vegetation, Main Res., DUF_100_	LC/SE	4	-57.17	122.39	62.43	2x10^-14^	13.4%
Sc1.5	GDD_0_, Rain_3w_, T_mean_10	M	4	-57.291	122.63	62.67	2x10^-14^	13.3%
Sc1.6	Vegetation, Main Res., Houses, DUF_300_	LC/SE	5	-56.543	123.16	63.19	10^−14^	14.4%
Sc1.7	GDD_10_, Rain_3w_, T_mean_10	M	4	-57.560	123.17	63.20	10^−14^	12.9%
Sc1.8	GDD_0_, Rain_3w_, DTR, T_min_10	M	5	-56.903	123.88	63.92	9x10^-15^	13.9%
Sc1.9	GDD_0_, Rain_3w_, T_max_10	M	4	-57.937	123.93	63.96	9x10^-15^	12.3%
Sc1.10	GDD_0_, Rain_3w_, T_max_7	M	4	-57.937	123.93	63.96	9x10^-15^	12.3%
Sc1.11	Vegetation, Main Res., DUF_300_	LC/SE	4	-57.952	123.95	63.99	9x10^-15^	12.3%
Sc1.12	GDD_10_, Rain_3w_, T_max_10	M	4	-58.101	124.25	64.28	8x10^-15^	12.0%
Sc1.13	GDD_10_, Rain_3w_, T_max_7	M	4	-58.101	124.25	64.28	8x10^-15^	12.0%
Sc1.14	GDD_10_, Rain_3w_, DTR, T_min_10	M	5	-57.176	124.43	64.46	7x10^-15^	13.4%
Sc1.15	GDD_0_, Rain_3w_, T_mean_7	M	4	-58.305	124.66	64.69	6x10^-15^	11.7%
Sc1.16	GDD_10_, Rain_3w_, T_mean_7	M	4	-58.424	124.90	64.93	5x10^-15^	11.5%
Sc1.17	GDD_0_, Rain_3w_, DTR T_min_7	M	5	-57.905	125.89	65.92	3x10^-15^	12.3%
Sc1.18	GDD_10_, Rain_3w_, DTR T_min_7	M	5	-58.076	126.23	66.26	3x10^-15^	12.1%

AICc: Aikake Information Criterion with a correction for small sample sizes; All: all categories of variables are included in the model (surveillance, meteorological, land cover and socioeconomic data); D^*2*^: explained deviance

LC/SE: only land cover and socioeconomic data are included as explanatory variables

M: only the meteorological data are included as explanatory variables; S: only the surveillance data are included as explanatory variables; Other variables used are provided in Table 4 and are detailed in S1.

We retained the global model Sc1.1 (Table 6) as the final model as it had the lowest AIC value among all models. The details of the other models, for scenarios Sc1 and Sc2, are provided in S3 Table.

**Table 6:** Results of binomial regression of autochthonous arboviral case emergence for model Sc1.1.

Variable	Coeff.	SE	z-value	OR (95% CI)	p-value
Intercept	-10.400	2.443	-4.257	3x10^-5^ (5x10^-8^–0.001)	<0.001
RD “long” #	2.964	0.820	3.615	19.4 (3.4–112.4)	<0.001
RD “missing” #	9.570	121.784	0.079	-	NS
GDD_0_	0.004	0.002	2.021	1.004 (1.001–1.009)	<0.05
Vegetation	0.047	0.017	2.765	1.05 (1.008–1.08)	<0.01

(#) “Short” RD (<21 days) as reference level. NS: non-significant

Explained deviance of the model: 62.2%

The RD of imported cases was the factor with the greatest explained deviance: between 48.9 and 52.8% according to the chosen scenario (Table 5). The proportion of variance explained was of the same order of magnitude for meteorological and land cover variables with values between 10 and 15% according to the specific model and scenario. Results of Sc1 and Sc2 were very similar. However, the sectoral analysis showed that explanatory variables related to land use were more sensitive to a lack of observations. For meteorological variables, no major difference was observed between the two constructions of GDD (i.e., at EDP and 10 days after EDP).

## Discussion

DENV and CHIKV are emerging threats in Europe. A better understanding of the respective contribution of the main determinants and drivers of emergence of these viruses is needed to identify most-at-risk situations, to prioritize interventions and, ultimately, to adopt a proactive surveillance scheme and implement an adequate public health response. To the best of our knowledge, this is the first comprehensive work exploring different determinants of DENV and CHIKV emergence in temperate settings using an epidemiological dataset.

Reporting-based failures constitute a major factor in the occurrence of autochthonous transmission. A delay in case identification has already been indicated as a contributing factor in contexts of extended viral circulation [58], and remains the most important factor for the occurrence of foci of limited transmission. Our current findings are consistent with our previous work based on a mechanistic approach [59]. Reporting delay is the combination of two elements: (1) how promptly the case-patient seeks medical care and (2) how responsive surveillance partners (e.g., medical analysis laboratories, hospitals, general practitioners, etc.) are in reporting cases. Any action which can positively impact one or the other of these two elements can contribute to the improvement of the arbovirosis surveillance system. Examples of such actions include the continuing effort to raise awareness of this surveillance system in healthcare professionals, the ongoing endeavour to consolidate a robust network of reporting diagnostic laboratories [8,19,29] and raising patient awareness of the importance of seeking medical consultation for non-specific febrile syndromes. The latter action however is more challenging as it is covers both the promotion of travel health consultation—which is not sought by the majority of tourists visiting at risk areas [60,61]—and the information provided to travellers in key locations such as airports. One avenue for research in this area would be to study whether socioeconomic determinants can explain delay or absence in seeking consultation [62], with a view to implementing targeted awareness-raising actions. Moreover, the surveillance system does not allow for the exhaustive detection of all imported viremic infections because of the existence of subclinical infections which are able to infect mosquitoes [63] and which may constitute the source of autochthonous transmission. This may partly explain why, for several autochthonous transmission events studied in France to date, a primary case could not be identified despite reinforced surveillance which carried out door-to-door surveys to identify cases [42]. Preparing for such a contingency (i.e., lack of primary case identification because of subclinical infections) requires maintaining a high level of vigilance so that any autochthonous transmission event can be promptly detected. Moreover, reducing the risk of transmission implies keeping vector populations as low as possible through encouraging the general public to reduce mosquito breeding sites, as larval control remains the main sustainable measure of prevention and control [27]. To a lesser extent, meteorological and environmental factors are associated with autochthonous transmission. The influence of temperature and consequently, the value of using Growing Degree Days (GDD) to describe vector population dynamics has already been reported in different European settings [36,64–66]. However, to the best of our knowledge, the present work constitutes the first time that this indicator has been used together with epidemiological data. GDD can be considered a proxy of the vector population density and reflects the importance of this parameter for estimating transmission risk. The presence of wooded areas is a landscape factor that could explain a favourable environment for *Ae*. *albopictus*. It is important to highlight that such areas were associated with anthropization in the present work, since they were located in the vicinity of the places where cases stayed. These green areas, or vegetated areas, are located either inside or are in direct proximity to peri-urban and residential areas which are known to provide suitable conditions for *Ae*. *albopictus* proliferation. Numerous rainwater collection containers dedicated to gardening are present and represent the most productive breeding sites for *Ae*. *albopictus* in temperate settings. Moreover, vegetation cover maintains relative humidity and provides shelter for adult mosquitoes to rest. Plant residues constitute a major food resource for mosquito larvae [67] and plant sugars are needed as an energy source for both male and female mosquitoes [68]. Finally, the presence of gardens, terraces and other green areas promotes outdoor living and therefore greater human exposure to the exophilic species that is *Ae*. *albopictus* [69].

Our study has limitations. We focused on the identification of drivers of dengue and chikungunya emergence. All events of autochthonous transmission were therefore considered, irrespective of their size. However, one may suppose that the factors explaining the size of the foci of transmission may be different from factors which only explain the occurrence of transmission. Furthermore, DENV and CHIKV were considered to be similar, despite differences in transmission efficacy. However, these vector-virus interactions are already highly heterogeneous for each of these pathogens due to the existence of different viral genotypes [70,71] and these two arboviruses exhibit a similar ecology calling for a similar public health response. These different considerations justify, in our view, treating both similarly. Furthermore, if we had had more events, we could have had studied the importance of virus genetic factors in more detail. Host and viral genetic factors may have a major impact on arbovirus transmission, as illustrated by the increased efficacy of transmission observed after the adaptation of chikungunya virus to *Ae*. *albopictus* [72]. The results of this work could be extended to the risk of transmission of other arboviral diseases, especially Zika, as the first case of autochthonous vector-borne transmission was recently reported in France [73]. Only events detected by the national surveillance system are reported here, and we cannot exclude that autochthonous transmission events—particularly of modest size—may have gone unnoticed, due to the absence of symptoms, medical consultation, proper diagnosis and reporting. The subclinical infection rates of dengue and chikungunya infections is also a possible limitation of the study, as the real incidence rates of imported cases were probably higher than those observed. Accordingly, the different risk factors identified were potentially overestimated. However, we assume that the impact of under-detection and under-diagnosis was limited given the efficiency of the surveillance system dedicated to dengue and chikungunya infections. *Aedes albopictus* is still expanding its range throughout the world, including Europe. However, it is difficult to extrapolate our results to other bioclimatic zones. It is therefore essential to update this type of approach in the light of new transmission events and in different environmental and climatic settings. Moreover, the presence of nested data (as a unique case introduction could lead to several observations) would have justified the use of mixed methods, but the limited number of transmission events did not contain any sufficient information for parameters estimation in such a framework. This limited number of transmission events is explained by the epidemiological situation and requires to consider the results with caution. In this global perspective, both routine documentation of viremic cases (from a meteorological, environmental, socio-economic point of view) and routine integration of data into surveillance system databases is paramount. The development of these anticipatory tools will be useful to reduce the risk of multiple autochthonous arboviral transmission events.

## Supporting information

S1 Table Description of the different explanatory variables. (DOCX)

S2 Table Results of binomial regression of autochthonous arboviral case emergence for scenario Sc2. (DOCX)

S3 Table Results of binomial regression of autochthonous arboviral case emergence for the different global multivariate models. (DOCX)

S1 Appendix Management of missing data of the reporting delay. (DOCX)
