# Supplementary material for: From importation to autochthonous transmission: Drivers of chikungunya and dengue emergence in a temperate area
Source: PLoS Negl Trop Dis. 2020 May 11;14(5):e0008320. doi: 10.1371/journal.pntd.0008320 (PMC7266344; doi:10.1371/journal.pntd.0008320)
Supplement: S1 Table — (DOCX) [file pntd.0008320.s001.docx]

## S1 Table. Description of the different explanatory variables

| **Meteorological variables** | |
| --- | --- |
| DTR | Weekly diurnal temperature ranges (DTR) after the earliest date of presence of an imported case during its viremia in the study area (EDP) |
| GDD_10_ | Bounded accumulated Growing Degree Days (GDD) with a baseline temperature of 11°C and a maximum threshold of 1350°C calculated from 1^st^ March of the year until 10 days after the earliest date of presence of an imported case during its viremia in the study area (EDP) |
| GDD_0_ | Bounded accumulated Growing Degree Days (GDD) with a baseline temperature of 11°C and a maximum threshold of 1350°C calculated from 1^st^ March of the year until the earliest date of presence of an imported case during its viremia in the study area (EDP) |
| Rain3w | Weekly cumulative rainfall 3 weeks before the earliest date of presence of an imported case during its viremia in the study area (EDP) |
| Rain2w | Weekly cumulative rainfall 2 weeks before the earliest date of presence of an imported case during its viremia in the study area (EDP) |
| Rain1w | Weekly cumulative rainfall 1 week before the earliest date of presence of an imported case during its viremia in the study area (EDP) |
| T_max_7 | Average maximum temperature over the 7 days after the earliest date of presence of an imported case during its  viremia in the study area (EDP) |
| T_mean_7 | Average temperature over the 7 days after the earliest date of presence of an imported case during its viremia in the study area (EDP) |
| T_min_7 | Average minimum temperature over the 7 days after the earliest date of presence of an imported case during its viremia in the study area (EDP) |
| T_max_10 | Average maximum temperature over the 10 days after the earliest date of presence of an imported case during its viremia in the study area (EDP) |
| T_mean_10 | Average temperature over the 10 days after the earliest date of presence of an imported case during its viremia in the study area (EDP) |
| T_min_10 | Average minimum temperature over the 10 days after the earliest date of presence of an imported case during its viremia in the study area (EDP) |

| Case surveillance and management variables | |
| --- | --- |
| Day of the year | Calendar day |
| Intervention delay | Delay (in days) between EDP* and entomological prospections and/or vector control. |
| Length of viremia | Estimated number of days during which the case is viremic in the study area. Viremia is considered to start one day before symptom onset and to end seven days after symptom onset. |
| RD | Reporting delay. Time (in days) between EDP* and the date of case reporting. |
| RDC | Reporting delay (cf. RD) as a categorical variable.  RDC = “short to medium” for RD between 0 and less than 21 days.  RDC = “high” for RD ≥ 21 days.  RDC = “missing” for RD = NA. |
| Sampling delay | Delay (in days) between EDP* and biological sampling |

*EDP: earliest date of presence of an imported case during its viremia in the study area

| Environmental and socieconomic variables (spatial variables) | |
| --- | --- |
| Buildings | Number of buildings within a radius of 300 m |
| Continuous urban fabric within a radius of 300 m | Percentage of continuous urban fabric within a radius of 300 m.  Continuous urban fabric is defined as follows: “Most of the land is covered by structures and the transport network. Buildings, roads and artificially surfaced areas cover more than  80% of the total surface. Non-linear areas of vegetation and bare soil are exceptional”. |
| DUF100 | Percentage of discontinuous urban fabric within a radius of 100 m. Discontinuous urban fabric is defined as follows: “Most of the land is covered by structures. Building, roads and artificially surfaced areas associated with vegetated areas and bare soil, which occupy discontinuous but significant surfaces. Building, roads and artificially surfaced areas cover less than 80% of the total surface” |
| DUF300 | Percentage of discontinuous urban fabric within a radius of 300 m. |
| Vegetation | Percentage of wooded areas within a radius of 300 m. The term Vegetation includes the different classes of closed and open forests of coniferous and deciduous tree species. |
| Main Res. | Percentage of main residences (versus secondary residences) at the IRIS* scale |
| NDVI within a radius of 300 m | Normalized difference vegetation index (NDVI) within a 300 m buffer around georeferenced cases. |
| Percentage of families among households | Number of households with family (couple with or without children), divided by the total number of households at the IRIS* scale |
| Percentage of houses (vs. apartments) | Percentage of house-type residences (compared with apartment-type residences) at the IRIS* scale |
| Percentage of vacant residences | Number of vacant dwellings divided by the total number of dwellings at the IRIS* scale |

* IRIS is an acronym for “aggregated units for statistical information”. IRIS are defined with a target size of 2000 inhabitants per basic unit and constitute the smallest geographical unit for which population census data with housing and socioeconomic details are available in France.
