# Supplementary material for: From importation to autochthonous transmission: Drivers of chikungunya and dengue emergence in a temperate area
Source: PLoS Negl Trop Dis. 2020 May 11;14(5):e0008320. doi: 10.1371/journal.pntd.0008320 (PMC7266344; doi:10.1371/journal.pntd.0008320)
Supplement: S2 Table — (DOCX) [file pntd.0008320.s002.docx]

## S2 Table. Results of binomial regression of autochthonous arboviral case emergence for scenario Sc2.

| Model | Variables | Variable category | df | logLik | AICc | delta | weight | D² |
| --- | --- | --- | --- | --- | --- | --- | --- | --- |
|  | Global multivariate models |  |  |  |  |  |  |  |
| Sc2.1 | RD, Vegetation, GDD_0_ | All | 5 | -24.9451 | 59.97 | 0.00 | 0.68 | 56.78% |
| Sc2.2 | RD, Vegetation, GDD_10_ | All | 5 | -25.7453 | 61.57 | 1.60 | 0.31 | 55.39% |
|  | Multivariate sectoral models | | |  |  |  |  |  |
| Sc2.3 | RD | S | 3 | -31.2044 | 68.44 | 8.47 | 0.010 | 45.93% |
| Sc2.4 | GDD_0_, Rain_3w_, T_max_10 | M | 4 | -49.7842 | 107.62 | 47.65 | 3x10^-11^ | 13.74% |
| Sc2.5 | GDD_0_, Rain_3w_, T_max_7 | M | 4 | -49.7842 | 107.62 | 47.65 | 3x10^-11^ | 13.74% |
| Sc2.6 | GDD_0_, DTR, Rain_3w_, T_mean_10 | M | 5 | -48.9307 | 107.94 | 47.97 | 3x10^-11^ | 15.22% |
| Sc2.7 | GDD_10_, Rain_3w_, T_max_10 | M | 4 | -49.9894 | 108.03 | 48.06 | 3x10^-11^ | 13.38% |
| Sc2.8 | GDD_10_, Rain_3w_, T_max_7 | M | 4 | -49.9894 | 108.03 | 48.06 | 3x10^-11^ | 13.38% |
| Sc2.9 | GDD_0_, DTR, Rain_3w_, T_min_10 | M | 5 | -48.9767 | 108.03 | 48.06 | 3x10^-11^ | 15.14% |
| Sc2.10 | GDD_10_, T_mean_10, Rain_3w_ | M | 4 | -50.2399 | 108.53 | 48.56 | 2x10^-11^ | 12.95% |
| Sc2.11 | GDD_10_, DTR, Rain_3w_, T_min_10 | M | 5 | -49.2502 | 108.58 | 48.61 | 2x10^-11^ | 14.66% |
| Sc2.12 | Vegetation, Main Res., DUF_100_ | LC/SE | 4 | -50.4897 | 109.03 | 49.06 | 2x10^-11^ | 12.51% |
| Sc2.13 | GDD_0_, DTR, Rain_3w_, T_min_7 | M | 5 | -49.7017 | 109.48 | 49.51 | 10^-11^ | 13.88% |
| Sc2.14 | GDD_0_, DTR, Rain_3w_, T_mean_7 | M | 5 | -49.7017 | 109.48 | 49.51 | 10^-11^ | 13.88% |
| Sc2.15 | GDD_10_, DTR, Rain_3w_, T_mean_7 | M | 5 | -49.8899 | 109.86 | 49.89 | 10^-11^ | 13.55% |
| Sc2.16 | GDD_10_, T_min_10, DTR, Rain_3w_ | M | 5 | -49.8899 | 109.86 | 49.89 | 10^-11^ | 13.55% |
| Sc2.17 | Vegetation, Main Res., Houses DUF_300_ | LC/SE | 5 | -50.0847 | 110.25 | 50.28 | 8x10^-12^ | 13.22% |
| Sc2.18 | Vegetation, Main Res., DUF_300_ | LC/SE | 4 | -51.2109 | 110.47 | 50.51 | 7x10^-12^ | 11.26% |

AICc: corrected Aikake Information Criterion; All: all categories of variables are included in the model (surveillance, meteorological, land-cover and socio-economic data); D²: explained deviance;
LC/SE: only the land cover and socioeconomic data were included as explanatory variables;
M: only the meteorological data were included as explanatory variables; S: only the surveillance data were included as explanatory variables. Other variables used are provided in Table 4 (main text) and detailed in S1.
