# Supplementary material for: From importation to autochthonous transmission: Drivers of chikungunya and dengue emergence in a temperate area
Source: PLoS Negl Trop Dis. 2020 May 11;14(5):e0008320. doi: 10.1371/journal.pntd.0008320 (PMC7266344; doi:10.1371/journal.pntd.0008320)
Supplement: S3 Table — (DOCX) [file pntd.0008320.s003.docx]

## S3 Table. Results of binomial regression of autochthonous arboviral case emergence for the different global multivariate models

| Scenario | Model | Variable | | Coeff. | SE | z-value | p-value | OR (95% CI) |  |
| --- | --- | --- | --- | --- | --- | --- | --- | --- | --- |
| Scenario Sc1 | Sc1.1 |  | (GDD0, Vegetation, RD) | | | | | | |
|  |  | **Intercept** | | -10.400 | 2.443 | -4.257 | 2e-5 | 3x10^-5^ (5x10^-8^-0.001) |  |
|  |  | **Reporting delay “long” #** | | 2.964 | 0.820 | 3.615 | 0.0003 | 19.4 (3.4-112.4) |  |
|  |  | **Reporting delay “missing” #** | | 9.570 | 121.784 | 0.079 | 0.937 | - |  |
|  |  | **GDD_0_** | | 0.004 | 0.002 | 2.021 | 0.043 | 1.004 (1.001-1.009) |  |
|  |  | **Vegetation** | | 0.047 | 0.017 | 2.765 | 0.006 | 1.05 (1.01-1.08) |  |
|  | Sc1.2 |  | (GDD10, Vegetation, RD) | | | | | | |
|  |  | **Intercept** | | -9.927 | 2.198 | -4.515 | 6e-6 | 5x10^-5^ (2x10^-7^-0.002) |  |
|  |  | **Reporting delay “long” #** | | 2.982 | 0.827 | 3.607 | 0.0003 | 19.7 (3.5-116.5) |  |
|  |  | **Reporting delay “missing” #** | | 9.084 | 121.537 | 0.075 | 0.940 | - |  |
|  |  | **GDD_10_** | | 0.003 | 0.002 | 1.991 | 0.046 | 1.003 (1.001-1.008) |  |
|  |  | **Vegetation** | | 0.049 | 0.017 | 2.834 | 0.005 | 1.05 (1.01-1.08) |  |
| Scenario Sc2 | Sc2.1 |  | (GDD0, Vegetation, RD) | | | | | | |
|  |  | **Intercept** | | -10.400 | 2.443 | -4.257 | 2e-5 | 3x10^-5^ (5x10^-8^-0.001) |  |
|  |  | **Reporting delay “long” #** | | 2.964 | 0.820 | 3.615 | 0.0003 | 19.4 (3.4-112.4) |  |
|  |  | **Reporting delay “missing” #** | | 9.412 | 151.329 | 0.062 | 0.950 | - |  |
|  |  | **GDD_0_** | | 0.004 | 0.002 | 2.021 | 0.043 | 1.004 (1.001-1.009) |  |
|  |  | **Vegetation** | | 0.047 | 0.017 | 2.765 | 0.006 | 1.05 (1.01-1.08) |  |
|  | Sc2.2 |  | Scenario Sc2, Model (GDD10, Vegetation, RD) | | | | | | |
|  |  | **Intercept** | | -9.927 | 2.198 | -4.515 | 6e-6 | 5x10^-5^ (2x10^-7^-0.002) |  |
|  |  | **Reporting delay “long” #** | | 2.982 | 0.827 | 3.607 | 0.0003 | 19.7 (3.5-116.5) |  |
|  |  | **Reporting delay “missing” #** | | 8.791 | 159.898 | 0.055 | 0.956 | - |  |
|  |  | **GDD_10_** | | 0.003 | 0.002 | 1.991 | 0.046 | 1.003 (1.001-1.008) |  |
|  |  | **Vegetation** | | 0.049 | 0.017 | 2.834 | 0.005 | 1.05 (1.01-1.08) |  |
