# Supplementary material for: From importation to autochthonous transmission: Drivers of chikungunya and dengue emergence in a temperate area
Source: PLoS Negl Trop Dis. 2020 May 11;14(5):e0008320. doi: 10.1371/journal.pntd.0008320 (PMC7266344; doi:10.1371/journal.pntd.0008320)
Supplement: S1 Appendix — (DOCX) [file pntd.0008320.s004.docx]

## S1 Appendix. Management of missing data of the reporting delay

The following options were considered:

- Option 1: only values recorded prior to the identification of the autochthonous focus retained;
- Option 2: values recorded after identification of the outbreak introduced;
- Option 3: all missing values and delays exceeding 21 days set at 21 days;
- Option 4: this scenario is similar to that of scenario 3, but the variable is converted to a categorical variable. Delay in [0;21[ as “short to medium” and delay ≥ 21 days as “high”;
- Option 5: all missing data (including reports of imported cases after autochthonous circulation) classified in a dedicated group, “missing” group.

**Table.** Results of univariate binomial regression model for the impact of different reporting delay options on the risk of autochthonous arbovirus transmission

| **Option** | **Estimate** | **AICc** | **delta** | **weight** | **D²** | **Nb** | **p-value** |
| --- | --- | --- | --- | --- | --- | --- | --- |
| Option 1 | 0.042 | 70.0 | - | - | 7.6 % | 6 | 3x10^-3^ |
| Option 2 | 0.049 | 84.2 | - | - | 19.6 % | 9 | 7x10^-9^ |
|  | | | | | | | |
| Option 5 | + | 70.6 | 0 | 1 | 52.1 % | 13 | 4x10^-3^ |
| Option 4 | + | 104.6 | 34.0 | 4.1e-08 | 25.3 % | 13 | 1x10^-7^ |
| Option 3 | 0.047 | 114.7 | 44.1 | 2.7e-10 | 17.8 % | 13 | 2x10^-9^ |

na: Comparison is not appropriate as models are not fitted to the same data.
AICc: corrected Aikake Information Criterion. D²: explained deviance.
Nb: number of transmission events included in the dataset.
